# Novel insights into the biology of childhood arthritis- lessons learned from the synovium

**DOI:** 10.1186/s13075-025-03717-2

**Published:** 2025-12-20

**Authors:** Chrissy Bolton, Margaret H. Chang

**Affiliations:** 1 Kennedy Institute of Rheumatology, University of Oxford, Oxford, UK; 2 Department of Inflammation and Ageing, University of Birmingham, Birmingham, UK; 3 Infection, Immunity and Inflammation Research and Teaching Department, University College London (UCL) Great Ormond Street Institute of Child Health, London, UK; 4 Division of Immunology, Boston Children’s Hospital, Boston, MA USA

**Keywords:** Juvenile idiopathic arthritis, Synovium, Flare, Tissue, Inflammation

## Abstract

Chronic inflammatory arthritis of childhood, known as juvenile idiopathic arthritis (JIA), exhibits distinct and shared features with adult-onset disease. Recent advances in minimally invasive synovial biopsy, high resolution imaging and sequencing technologies have enabled detailed characterisation of the inflamed synovium in JIA, facilitating insights into the underlying immunopathology. In this review we draw on these findings to consider how the developing immunological and structural context of the joint impacts the presentation and consequence of inflammatory joint disease in children and young adults.

## Introduction

Juvenile Idiopathic Arthritis (JIA) is an immune-mediated, non-infectious form of arthritis that affects two million children globally [1]. Characterised by chronic inflammation of the synovial joint lining, it causes pain, stiffness, and fatigue, which significantly impacts the daily life of children affected by the disease [2]. Clinical management itself places substantial burden on children and their families due to debilitating drug side effects, frequent injections and regular therapeutic monitoring [3], but if chronic inflammation is not adequately controlled, irreversible joint damage and disability will result [4, 5]. Despite these challenges, there is hope, as children with JIA are more likely to achieve drug-free remission than adults with rheumatoid arthritis (RA) [6, 7]. In this review, we consider how unique aspects of the developing joint—including its structural, immunological and physiological context—may influence the outcomes and consequences of joint inflammation in JIA.

### The developing joint structure in childhood and adolescence

Almost all the joints affected by childhood-onset arthritis are synovial joints [8], defined as highly movable joints formed with a lubricated cavity between the articulation of bones. By the time of birth, the main components of synovial joint structures have already formed [9]: opposing ends of bones are lined with the more compressible articular cartilage, a secretory synovial membrane encircles non-articulating regions, and a strong fibrous capsule stabilises bones together [10]. By birth, most of the cartilage template of the bony skeleton has undergone replacement by bone, a process known as ossification [11]. At the rounded ends of long bones, cartilage continues to proliferate in the growth plate (also known as the epiphyseal plate) and ossify, increasing bone length and reducing cavity space [12, 13]. This typically continues until the end of puberty, when the cartilage becomes completely replaced with bone (epiphyseal fusion) [12].

The articular cartilage lining the bone surface expands with growth, maturing from a highly cellular tissue with scanty matrix at birth, to an highly organised structure that is dense with extracellular matrix [12]. The reparative capacity of the articular cartilage with age is limited by its avascular and highly specialized nature [14]. Increased plasticity and regenerative capacity in childhood and adolescence has been attributed to the greater vascularity of the underlying unfused growth plate, less calcification of layers within the articular cartilage and greater proliferative potential of progenitors [14–16].

The synovial membrane, which provides joint lubrication, is the primary site of pathology in inflammatory arthritis. In health, it is formed of a barrier-like lining layer of fibroblasts and resident macrophages, which interface the joint cavity space, taking up debris; and a looser sublining layer below, primarily composed of fibroblasts, lymphatics, vessels and adipocytes [17–19]. Human studies describing how the healthy synovium changes with age are scarce. Historical studies based on small numbers suggest some developmental distinctions. At birth, few villi are present (finger-like projections of the membrane), which increase in number with age and disease [9, 17]. From adolescence onwards, vessel number does not appear to change substantially [20], though the vascular network becomes less linear and more irregular. Synovial adipocytes, which are particularly abundant in weight-bearing joints [10], have been seen to decrease from adolescence to adulthood, whereas mast cells and macrophages increase during this period [20]. Overall, a greater understanding of the healthy synovium in children is needed to better contextualise and interpret disease changes.

### Structural changes in the synovial membrane in childhood arthritis

**Fig. 1:**
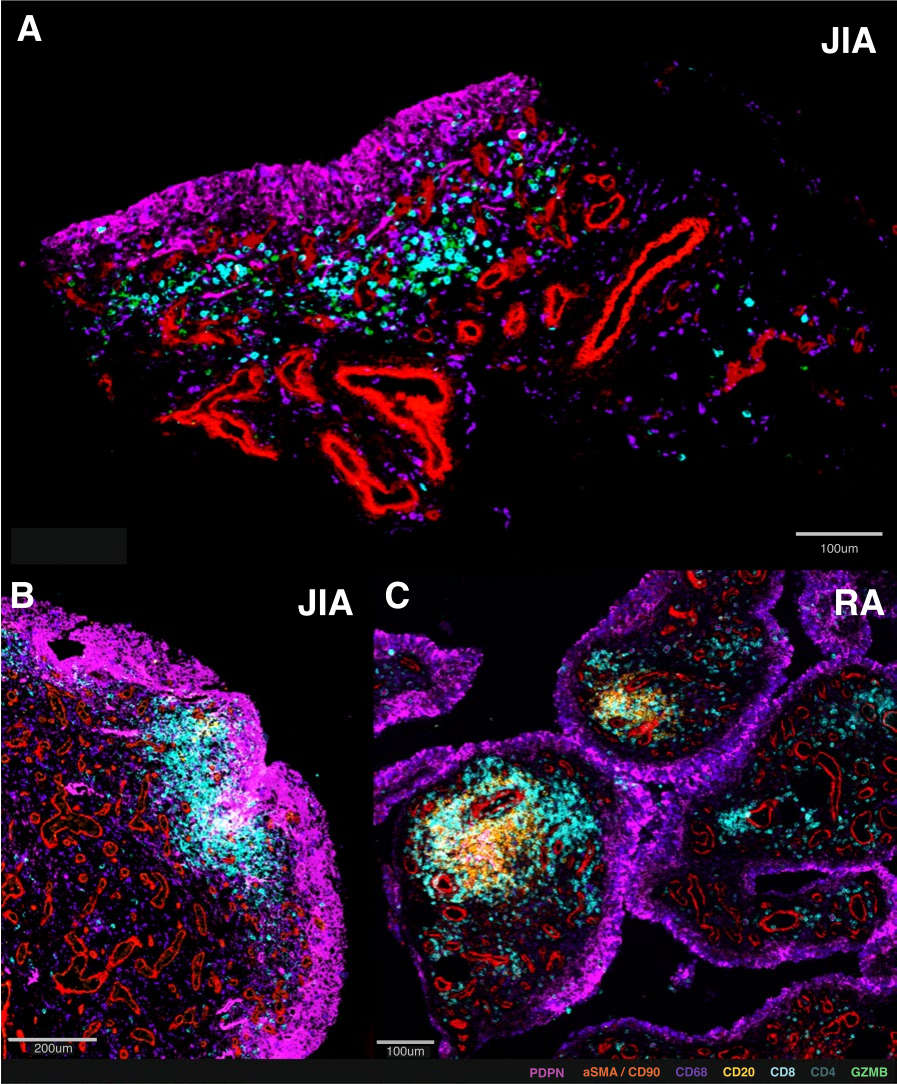
Lymphoid aggregation in inflamed synovial tissue from adults and children with inflammatory arthritis. **A-C** Multiplexed immunofluorescence staining showing T cells (cyan) and B cells (yellow) approximating the lining layer (pink) in synovial tissue from children with nsJIA (**A-B**) and adults with seropositive RA (**C**), adapted from analyses performed in ref [30, 36]. Immunofluorescence markers indicated in colour key below. nsJIA = non-systemic Juvenile Idiopathic Arthritis; RA = rheumatoid arthritis

In inflammatory arthritis of children and adults, the synovial membrane thickens with an increase in the lining and sublining layer fibroblasts, infiltration of immune cells from the circulation, and development of new vessels [17, 21, 22] (Fig. 1A). The membrane can form an invasive mass, known as a pannus, which erodes into underlying cartilage and bone where they join with the synovium. Early in disease, activation of the clotting cascade in the synovial cavity leads to deposition of insoluble fibrin clots, which may become subsumed into the membrane [23, 24]. Immune cells aggregate within the synovial sublining, largely at the interface between the lining and sublining layers, while the vascular layers with larger vessels lie deeper within the synovial sublining. Despite similarities between presentations of inflammatory arthritis in children and adults (Table 1), synovial composition highlights shared and distinct pathogenic features that may be exploited in therapeutic targeting [26, 30, 35].

**Table 1:** Overlaps in the clinical classification of childhood- and adult-onset arthritis

JIA encompasses many subtypes of disease historically classified by their clinical features, including joint count and autoantibody development [25]. Comparable disease subtypes between paediatric and adult-onset arthritis have been highlighted in recognition of the overlap in associated features and genetic susceptibility [26–28]. Namely, seropositive polyarticular JIA resembles adult seropositive RA; systemic JIA and Adult Onset-Still’s disease are now collectively recognized as one disease [29]; and enthesitis-related arthritis, spondyloarthritis and psoriatic arthritis share genetic, clinical and mechanistic overlap [26, 27]. Oligoarticular JIA and seronegative polyarticular JIA, the most prevalent subtypes in JIA, have been linked to seronegative RA and exhibit some shared genetic suceptibility [26]. Historically, this group has been split according to the number of joints affected (oligoarticular vs polyarticular JIA) [25], but more recently a division has been proposed based on early disease onset in combination with the formation of anti-nuclear antibodies (early-onset ANA-positive JIA) [28]. Clinical classifications have provided a useful framework for broadly classifying disease, but the advent of high-resolution profiling technologies, together with advances in minimally invasive synovial biopsy, has enabled characterisation of synovial pathology with unprecedented detail [30, 31]. These approaches have demonstrated marked cellular and molecular heterogeneity within disease groups connected to variability in therapeutic response in adults [32, 33]. Although synovial biopsies from the knees of children appear to be more homogeneous than those from adults [30], the variability in treatment response is nonetheless indicative of important underlying differences in pathology [34].	

#### Vasculature

Our understanding of human arthritic synovial tissue has largely been inferred from studies of adult RA, with new high-resolution technologies providing novel insights into the finer architecture of pathogenic cell types present in tissue [18, 33]. The first single-cell RNA-sequencing (scRNA-seq) atlas of JIA synovium was recently published [30], which conducted comparative analyses of arthritic synovium between adults and children, matched for disease duration, treatment exposure and joint site. Although new vessel formation and synovial hypervascularity are hallmarks of joint inflammation [17], the degree of vascularity was proportionally higher in children with non-systemic (ns) JIA compared to adults with RA [30]. Specifically, scRNA-seq of synovial tissue from children showed a higher proportion of pericytes, venous, capillary and lymphatic cells than adults, confirmed with multiplexed immunofluorescence imaging [30]. In a parallel study, spatial transcriptomic analysis similarly confirmed enrichment of *NOTCH3* + sublining fibroblasts (perivascular cells) in nsJIA synovium [37], alluding to increased vascularity compared to RA, which was evident irrespective of the greater heterogeneity in treatment use, disease duration and joint site in the analysed cohorts [38]. It is worth noting, given the known association with abnormal tortuous vessel formation in adult-onset spondyloarthropathies [39], that spondyloarthropathy subtypes made up a small proportion of these nsJIA cohorts. In addition, histological observations identified pronounced synovial vascularity in both juvenile spondyloarthritis and oligoarticular JIA compared to adult-onset disease forms [40]. Intriguingly, mesenchymal stem cells (connective tissue progenitors) from children display enhanced angiogenic responses to stimuli [41], providing a potential mechanism for this observation.

The functional consequences of increased vascularity and lymphatic formation in this context have not been fully determined. Increased synovial vasculature promotes immune cell infiltration and is linked to greater drug penetration [42]. However the structural irregularities and altered permeability of these rapidly formed vessels [43] may impair their function, as seen in malignant solid tumours where drug penetration across the full depth of tissue is impeded by these characteristics [44]. Lymphatic vessels facilitate drainage of inflammatory cells from the tissue to lymph nodes, reducing cell accumulation and aggregation in the membrane itself [45]. Chronic inflammation in animal models leads to loss of contractility in these vessels and reduced cellular trafficking [45], raising the question of whether this contributes to differences in lymphoid aggregation across the age spectrum, which we discuss further in the ‘B cell and plasma cell’ section.

#### Fibroblasts

The architecture of the synovium is driven through a combination of biomechanical forces via mechanosensors (i.e. PIEZO1/2 channels) and signalling gradients such as NOTCH3 [37, 46]. Fibroblasts within the lining layer mediate bone and cartilage damage, the invasive and erosive features of a synovial pannus, while Thy1 + sublining fibroblasts contribute more to perpetuating inflammation with cytokine production, immune cell chemotaxis, complement activation and vasculogenesis [47]. Amongst fibroblast states, a TGF-β driven *POSTN* + subset was enriched in nsJIA synovial sublining compared to knee biopsies from adult samples [30]. The abundance of this population negatively correlated with age, being highest in the youngest participants with nsJIA. These perivascular fibroblasts display an expression profile consistent with synthetic remodelling through collagen production/organisation [30, 36]. Evidence against a purely homeostatic developmental role comes from genome-wide association studies that find a more severe disease course associated with different alleles in the *POSTN* gene [48]. Extracellular matrix production by *POSTN* + fibroblasts is associated with immune exclusion in cancer and adult arthritis [36, 49], raising the question of whether remodelling of collagen around vessels by this cell state contributes to the high rates of drug-free remission observed in early-onset arthritis [50].

#### Adipocytes

Little is known about the role of synovial adipocytes in the developing joint. In adults and children, synovial adiposity is a feature of non-inflamed synovium [30, 51]. In adults, inflammatory remodelling of the joint is accompanied by a reduction in synovial adiposity, which associates with disease progression [51]. In vitro studies further support a protective function in the local context of the tissue, whereby adipose-conditioned media induces a “healthy” fibroblast signature in RA fibroblasts [19]. The inflammatory setting and anatomical context of adiposity may exert distinct effects, since the periarticular fat pads have been identified as a rich source of bioactive mediators that can drive pro-inflammatory TNFa and IL-6 pathways, and promote cartilage degradation [52, 53]. Equally, in animal models, adipose tissue has been shown to harbour and expand pathogenic CD8 + T cells that express high levels of IFNα, which can promote inflammation on trafficking to arthritic joints [54]. Indeed, systemic obesity correlates with increased arthritis severity and is particularly implicated in psoriatic disease where risk of transition to arthritis and treatment response is directly associated [55, 56]. In Western cohorts, around a third of children with psoriatic JIA are overweight, suggesting this pathogenic link is common to juvenile forms as well [57]. Overall, obesity rates are higher in adults than children but have continued to rise in all age groups over time [58]. As more children become obese, these extra-articular reservoirs of inflammatory mediators may increasingly impact arthritis severity.

### Consequences of arthritis on the articular cartilage in children

As in adult-onset forms of arthritis, the articular cartilage can become damaged by inflammation within the joint lining. Inflammatory cytokines within the inflamed synovial fluid such as IL-1 and TNF stimulate cartilage cells (chondrocytes) to secrete metalloproteases, and these matrix degrading enzymes break down the cartilage [59]. Further, the inflamed pannus can directly invade into cartilage and bone, eroding the articular surface and adjacent structures [21]. In children, even joints that have not been directly affected by arthritis display thinner articular cartilage than healthy children [60]. This points to a global dysregulation of cartilage homeostasis, potentially as a consequence of systemic inflammation. Detachment of bone from the articular surface (osteochondritis dissecans) is a rare pathology typically seen in adolescents, but has been observed in young children with JIA [61], reflecting enhanced structural vulnerabilities from bone demineralisation or interruptions to vascular supply.

There is greater plasticity in the developing cartilage of children, with increased use shown to enhance thickness during adolescence [62]. Cartilage-producing chondrocytes derive from progenitors residing in the articular cartilage, but mesenchymal stem cells in synovial fluid can also give rise to chondrocytes [63]. These display a greater proliferative and synthetic capacity in children than adults, producing higher volumes of extracellular matrix [15, 16, 64]. However in children with JIA, mesenchymal stem cells display impaired ability to differentiate into cartilage-producing cells, self-renew or repair cartilage in vivo, even compared to adults with RA [63], illustrating that joint inflammation not only causes cartilage damage, but may also hinder reparative processes in children.

### Age related differences in the effect of arthritis on the developing bone

In children, the consequences of inflammation can impact the development of the joint and bone, causing asymmetric or stunted growth, and lead to a lifelong loss of bone mineral density. Leg length inequalities can arise as increased blood flow from inflammatory mediators locally drives overgrowth initially, followed by accelerated epiphyseal maturation, leading to premature closure of the growth plate (epiphyseal fusion) [13, 65]. In contrast, in arthritis of the temporomandibular joint, the close proximity of the mandibular growth plate to the condyle risks damage to the physis, leading to jaw undergrowth or micrognathia [66]. Because of the transitioning state of the developing skeleton, arthritis also produces different focal forms of bony damage in children from those seen in adults [13, 67]. Shape deformities of carpal bones in younger children may occur rather than discrete erosions because of the greater plasticity of the growing bones. However, this skeletal plasticity during development can be beneficial to children as well, as individuals with JIA who respond to methotrexate have corresponding improvements in bone formation while adults with RA whose skeletons are mature often continue to show progression of bony damage despite clinical improvement [68]. There is a limit to the extent to which developing bone can compensate for damage however, as children who do not respond to treatment will continue to accrue joint space narrowing and erosions.

The mechanisms and long-term consequences of global bone loss from arthritis are also impacted by age. Childhood and adolescence are key periods for the acquisition of bone mass, with exponential increases in bone mineral content during this period [69]. Reduced bone density in childhood increases the fracture risk in later life, and is observed in around half of adults with JIA [69]. In prepubertal children with JIA, bone formation is suppressed, whereas in older adolescents or adults with arthritis, bone thinning arises from enhanced bone resorption that increases the fracture risk [70]. Excessive osteoclast activation, immobility due to pain, strong systemic treatments, endocrine dysregulation and elevated pro-inflammatory cytokines are all considered to play a role [70].

### Age-related distinctions in immune composition of the inflamed synovium

#### B cells and plasma cells

The central role of autoantibody formation provides a key distinction in the pathogenesis of the most common disease forms in childhood- and adult-onset arthritis. The majority (~ 80%) of adults with RA are seropositive, meaning they produce autoantibodies against rheumatoid factor or cyclic citrullinated peptides, whereas seropositive polyarthritis accounts for only a small proportion (~ 5–10%) of individuals with JIA [71]. Central to the pathogenesis and progression of this subtype, autoantibodies generated by plasma cells contribute to immune complex formation, which activates innate immune cells and complement pathways, as well as promoting osteoclast differentiation and bone erosion [72]. Rheumatoid factor autoantibodies are classically of the IgM isoform, and this is reflected within tissue, where compared to adults with RA, IgM + plasma cells are scarce in the synovium of children with seronegative nsJIA [30].

In one-quarter of adults with long-standing RA, B cells are organised within the synovium in ectopic germinal centres [73]. These are specialised microstructures supported by a network of follicular dendritic cells, which facilitate T-B cell interactions to enhance B cell proliferation and maturation into plasma cells [73]. Remarkably, these ectopic germinal centres from humans can sustain ongoing pathogenic autoantibody production, even when transplanted into lymphocyte-deficient mice [74]. In contrast, mature germinal centres are rarely observed in JIA, even in long-standing disease. Instead, looser clusters of T and B cell aggregates predominate, which lack follicular dendritic cells [75] (Fig. 1B-C). This pattern does not merely reflect disease duration, as it remains stable over time in both children and adults. Rather, the formation of lymphoid aggregates correlates with the degree of plasma cell infiltration and anti-nuclear antibody formation in children, suggesting their presence may represent a distinct immunopathological phenotype [76].

In children with nsJIA, plasma cells infiltrating inflamed synovium are predominantly IgG + [30]. Peripheral blood mononuclear cells from children with JIA under six years of age, show enrichment for B cell and plasma cell signatures compared to older individuals with JIA [77], which is not seen in age-matched controls, suggesting a disease-specific effect. Other disease features including formation of anti-nuclear antibodies and concurrent uveal inflammation of the eye also correlate with younger age [28, 77]. Consistent with these findings, tissue-level data demonstrate increased plasma cell infiltrates in younger children, which localise to the sublining [30]. Whether these age-related distinctions reflect differences in the underlying pathogenesis or the immune context of early childhood is not known, but they do nonetheless highlight an opportunity for therapeutic targeting.

### T cells and innate lymphoid cells

The T cell compartment evolves with age in its cellular composition, phenotype and clonal diversity, transitioning from a tolerogenic, innate-skewed repertoire in early life to a memory-enriched, antigen-experienced population as it adapts to microbes and environmental factors over time [78–80]. These shifts are reflected in the spectrum of innate and adaptive lymphocyte subsets populating the inflamed synovium, with greater proportions of CD4 + and CD8 + memory T cellular subsets observed in adults with RA, and relative enrichment of unconventional and innate lymphocytes, including γδ T cells, Mucosal-Associated Invariant T cells (MAIT) and innate lymphoid cells in children [30, 81]. Studies of the healthy population and adults who were previously diagnosed with JIA suggest this is a distinction of age, rather than a disease-specific consequence [80, 81].

#### CD4 + T cells

In both adult and childhood-onset arthritis, CD4 + T cells are understood to be key drivers of disease pathogenesis. This is supported by the fact that the strongest genetic risk factors for arthritis reside within the HLA class II locus, which governs antigen presentation to CD4 + T cells, shaping their activation and differentiation [27]. Across the age spectrum, clonally expanded CD4 + memory T cells contribute to synovial inflammation by producing inflammatory cytokines, activating macrophages and fibroblasts, and stimulating B cells [82]. The synovial CD4 + T compartment is dominated by three main subsets: IFNγ-producing Th1 cells (which activate myeloid cells); fibroblast-stimulating Th17 cells; and T peripheral helper cells (Tph, PD-1hiCXCR5-), which augment B cell function [35, 83]. Of note, T follicular helper cells (PD-1 + CXCR5 +) that support B cell activity have also been described in RA synovium, but were notably not identified in recent studies of JIA synovium [30]. Genetic studies and therapeutic response patterns have delineated the role of distinct T helper cells in different disease subtypes. For example, IL-17-blockade showed greater efficacy in juvenile and adult-onset spondyloarthritis than adult RA [84]. Nonetheless a lack of clinical trials and synovial tissue studies have precluded definitive identification of which cell types are most influential in each subtype of JIA.

#### Regulatory T cells

In healthy children, CD4 + T cells show an age-dependent maturation of both inflammatory and immunoregulatory responses, with intracellular levels of pro-inflammatory cytokines (IFNγ and TNF) and immunosuppressive cytokines (IL-10) positively correlating with age [85]. Similar synovial proportions of *CD4* + *FOXP3* + regulatory T cells (Treg) have been observed in nsJIA and adult RA synovium [30], however in donor lymph nodes, expression of *FOXP3* was higher in Tregs in children than adults, suggesting enhanced functionality [86]. Across the age spectrum, Tregs can adapt their epigenetic and transcriptional profiles in the inflammatory environment of the arthritic joint, forming “effector” Treg states [87]. This has been attributed to secondary reprogramming in the inflammatory synovial environment, whereby IFN-induced Tregs attain Th1 features, IL-1β-induced Tregs acquire osteoclastogenic features, and IL-6-induced Tregs obtain Th17 features [83, 88–90]. Notably, these synovial Tregs maintain their suppressive capacity while sharing pro-inflammatory features. Understanding the dominant inflammatory context is therefore key for determining how to restore Treg fitness and re-establish immune tolerance.

#### CD8 + T cells

The distribution of T cells in the inflamed synovium predominantly follows a similar pattern to B cells, forming diffuse infiltrates or more organised aggregates, which associate with disease severity [22, 75, 76]. However in a third of children with nsJIA, a dense CD8 + T cell band is observed approximating the lining layer [76], which is not typically observed in adult RA; the significance of which is not well characterised. Amongst synovial CD8 + memory T cells, the majority express granzyme K (*GZMK),* which augments inflammation locally through cleavage of complement produced by fibroblasts [30, 91–93]. GZMK + CD8 + T cells are major producers of inflammatory cytokines such as IFNγ, but have low cytotoxic potential compared to the more well-studied cytotoxic CD8 + T cells that express granzyme B (*GZMB*) [93]. In nsJIA synovium, *GZMK* + *GZMB-* T cells formed the largest population of CD8 + T cells, whereas *GZMK* + *GZMB* + T cells were the most abundant in RA [30, 93]. This mirrors the acquisition of cytolytic profiles observed in NK cells and CD8 + T cells across many tissues with increasing age [94]. Tissue residency markers (for example CD103/*ITGAE*, CXCR6, CD49a/*ITGA1*) are expressed by GZMK +, Tph, Th17 and GZMB + memory T cells in the arthritic synovium, indicating a range of effector phenotypes may contribute to the joint-specific memory underlying the recurrence of inflammation in previously affected joints [30, 92, 95, 96].

#### Innate/-like lymphocytes

Whilst conventional T cells recognize peptide antigens presented by MHC molecules, unconventional or innate/-like lymphocytes recognize a broader range of substrates, including lipids, microbial ligands and modified metabolites [97, 98]. This group of lymphocytes possess more restricted or invariant T cell receptors than conventional T cells and can respond rapidly to stress and cytokine signals in the microenvironment [98]. Children have higher frequencies of VD1 + T cells in tissue than adults [99], and this remains true in the inflamed joints of children with nsJIA, where their tissue proportions followed an inverse correlation with age [30]. The VD1 + T cells possess an ‘activated NK-like’ phenotype, with expression of *KLRC2, AREG* and a lack of cytolytic markers (perforin or IFNγ), that most closely aligns with the ‘repair’ module described in other tissue contexts [99]. Of note, in contrast to studies of murine arthritis and synovial fluid samples, T cells and MAIT in synovial tissue from those with spondylarthropathies were recently shown to lack IL-17 expression compared to CD4 + T cells raising further questions of their role in arthritis [100].

### Myeloid cells

#### Monocytes and macrophages

Resident *MERTK* + synovial macrophages maintain tissue homeostasis through spatially and functionally specialized roles that are essential for establishing and sustaining remission in arthritic disease [18, 101]. Among these, *MERTK* + *LYVE1* + macrophages residing around vessels form a major population in health and remission [30, 102]. Whilst not fully characterised in human synovium, this population has been shown to support adipose niches, modulate perivascular collagen deposition in the lung and, conversely, facilitate monocyte recruitment in early stages of murine arthritis [103–105]. Closer to the synovial cavity, highly phagocytic *MERTK* + *TREM2* + lining layer macrophages form tight junctions that effectively seal off the synovium from the cavity, surveilling and clearing debris from the intervening space. Intriguingly, the *APOE-TREM2* signature characteristic of these lining layer macrophages has been shown to decline with age in myeloid cells across diverse tissue sites, suggesting changes in macrophage states could progressively compromise synovial integrity over time [94].

During active arthritis, monocytes infiltrate into the joint and differentiate into pro-inflammatory macrophages that secrete cytokines like TNF, IL-6 and IL-1β, thereby stimulating fibroblast proliferation, matrix metalloproteinase production and osteoclast differentiation, which together drive joint erosion [18]. In JIA, *IL1B* + and *SPP1* + macrophages are abundant in the inflamed synovium [30, 38]. Their developmental origins from circulating monocytes or resident populations have not been fully elucidated. Whilst both cell types have been characterised as pro-inflammatory populations that drive synovitis [18, 30], *SPP1* + macrophages have also been linked to tissue remodelling and fibrosis responses, suggestive of a pro-resolving function [106]. There is some evidence to suggest a prominent role for *IL1B* + macrophages in childhood arthritis, namely the increased prevalence of Still’s disease in children compared to adults, which is driven by excessive IL-1b signalling [107–109], and the enrichment of *IL1B* + myeloid cells in nsJIA synovium compared to adult RA [30, 38]. This enrichment of *IL1B* + myeloid cells does not appear to simply follow a linear relationship with age, as *IL1B* + myeloid cells proportionally increased from early childhood to teenage years. Further work is needed to provide finer resolution of myeloid cell phenotypes and their temporal roles going forward.

#### Dendritic cells (DC)

Dendritic cells are the most powerful antigen-presenting cells, directing immune responses by activating and guiding T cell fate towards regulatory or effector states. A resident DC2 population expressing *AXL* has been recently identified approximating the lining layer in healthy adults, which expresses tolerogenic markers, becomes diminished in inflammation and does not become re-established in sustained remission [110]. The presence of this DC state in children’s tissue has not been able to be confirmed given the scarcity of studies in this area. In the inflamed synovial microenvironment, where DC accumulate in greater numbers, *CLEC10A* + DC (DC2 and DC3 states) comprise the most abundant DC type, showing similar proportions in both JIA and RA [30, 91]. In synovial fluid from those with JIA, which may be more likely to capture migratory DC, a highly stimulatory and pro-inflammatory state has been highlighted amongst conventional DC2, as compared to the more quiescent conventional DC1 population [111]. Whilst DC2 are still detected in non-arthritic synovial samples, plasmacytoid DC, the key producers of IFN, are almost non-existent [30]. Plasmacytoid DCs have been shown to be specifically elevated in JIA compared to septic arthritis, suggesting a distinct role in autoimmunity [112]. Equally, healthy children display higher levels of plasmacytoid DC than adults, raising the question of how this impacts synovial inflammation that arises in childhood [112–114].

#### Neutrophils

Neutrophils influx into the synovium in early arthritis, where they amplify or regulate tissue damage through antigen presentation and the release of reactive oxygen species, proteolytic enzymes (which can degrade cytokines as well as extracellular matrix and complement components), and neutrophil extracellular traps [115]. Of particular relevance to the pathogenesis of seropositive arthritis, neutrophils express the peptidylarginine deiminase 4 (PADI4) enzyme implicated in generating citrullinated autoantigens that trigger autoantibody formation [115]. Their relevance to multiple arthritic forms across the age spectrum is suggested by their abundance in synovial fluid, where they comprise the majority of cells in those with oligoarticular JIA and adult RA [116–118]. Here they adhere to fibrin networks, impeding its breakdown, and associate with the incidence of morning stiffness [30, 116–118]. However, the fragility and short life span of these cells frequently limits their recovery in scRNA-seq datasets [116], complicating efforts to delineate age-dependent differences in their role across paediatric and adult disease.

#### Mast cells

Resident to the joint, mast cells are another granulocyte population implicated in early arthritis, which expand in the inflammatory environment of adults with RA [119]. They are found clustered around blood vessels and nerves in healthy synovium, and localise to the adipocyte-rich niches in JIA [30, 119]. Consistent with their distribution in relatively uninflamed regions, murine studies suggest they play a role in the early stages of arthritis, by initiating autoimmune-mediated synovitis in an IL-1-dependent manner [119]. However the paucity of human data, particularly in paediatric disease, precludes conclusions about their function in JIA.

### Factors impacting arthritis severity

Undeniably, of all JIA subtypes, seropositive JIA is associated with the most concerning long-term outcomes, with high rates of joint damage and poor rates of sustained remission [120, 121]. Across the other JIA subtypes, particularly oligoarticular JIA, ongoing research has focused on identifying predictors of disease course. Clinical markers associated with a more severe trajectory in oligoarticular JIA include elevated ESR or CRP levels, as well as involvement of upper-extremity joints, particularly wrists [8, 122]. Further, studies analysing synovial fluid have identified differences in lymphocyte subsets that correlate with a progression of disease to an increased number of joints. These include increased ratios of CD8 + T cells relative to CD4 + T cells, increased IFNγ-producing Th1 cells, higher proportions of Th17 cells and reciprocally lower Treg cells [83, 90, 123].

At the tissue level, a study of synovial tissue samples collected from individuals at the time of JIA diagnosis identified several stromal and immune features that correlate with more severe disease. Higher B cell and T cell infiltrates, particularly CD4 + T cells, in the synovium of new-onset JIA patients correlated with an extended or polyarticular course compared to those with persistent oligoarticular JIA. Equally, those with JIA who exhibited more fibrin deposits in the synovial lining were more likely to have suffered inadequate response to two or more biologic therapies [22, 24]. This was further confirmed by a recent spatial transcriptomic study of JIA synovium, which found that populations enriched in predictive markers for severe disease, namely *SPP1* + macrophages, localised to fibrin deposits [30]. Larger and more highly powered studies are needed to address how the tissue microenvironment reflects longitudinal outcomes in greater detail.

### Predictors and biology of arthritis flares

Investigators have long sought to identify markers that can predict arthritis flares, as the ability to anticipate flares could inform clinical decisions to taper or discontinue therapy (Table 2). However, until recently, little was known about the biology of arthritis flares.

**Table 2:** Predictors of arthritis flares

Clinical indicators [24, 124, 125]:
•Polyarticular disease
• Positive autoantibodies (RF +, ANA +)
• High levels of inflammatory markers (ESR, CRP) at diagnosis
• Higher disease activity score at diagnosis
• Shorter duration of clinically inactive disease
• Shorter duration since diagnosis of JIA
Biomarkers [18, 24, 95, 126–131]:
• Increased synovial hypertrophy at diagnosis
• Lower percentages of double negative and T cells in synovial fluid at diagnosis
• Elevated serum S100A12 and calprotectin levels during inactive disease
• Evidence of inflammation (i.e. increased power doppler signal, synovial hypertrophy or effusion) on ultrasound imaging during inactive disease

Longitudinal sampling of the peripheral blood of RA patients identified B cell activation followed by expansion of circulating preinflammatory mesenchymal cells one to two weeks preceding arthritis flares [130]. As circulating levels of these cells decreased during the flares themselves, it was hypothesized that these cells may have a pathogenic role in the escalation of inflammation. In a similar study of RA patients before and after flare, there was an increase in circulating activated memory B cells and T cells during flare. However, arthritis relapse was attributed to dysfunctional circulating Tregs with reduced CD39 and *IFITM2* expression found in flare patients [132].

More recently, studies of immune cells within the synovial tissue have led to breakthroughs in our understanding of the biology underlying arthritis flare. Resident memory T (T_RM_) cells are T cells that persist in local tissues after inflammation and provide long-term immune protection. T_RM_ cells have been identified in the synovial tissues of RA, spondyloarthritis and psoriatic arthritis patients by microscopy and RNA sequencing [96]. Similar T_RM_-like cells have also been identified in JIA synovial fluid [96]. Antigen-specific activation of synovial T_RM_ cells led to arthritis flares by recruiting effector immune cells, while localized depletion of T_RM_ cells in remission attenuated recurrent disease [95]. In addition to T_RM_ cells, synovial tissue macrophages have also been implicated in arthritis relapse. Resident *MERTK* + macrophages exhibit a regulatory phenotype that supports tissue integrity and immune tolerance while monocyte-derived *MERTK-* macrophages are suggested to be pro-inflammatory. A lower proportion of resident *MERTK* + macrophages in RA synovium during remission has been associated with increased risk of arthritis flare after treatment discontinuation [18].

## Conclusion

Understanding how age influences inflammation in arthritis is essential for disentangling the developmental, immune, and tissue-specific factors that shape disease onset and progression. Age affects both the composition and reactivity of immune and stromal cells, altering cytokine networks, repair mechanisms, and the balance between innate and adaptive immunity. Yet a detailed understanding of tissue immunology during development remains a significant knowledge gap in human biology, with a paucity of comparative studies and high-dimensional datasets that incorporate children and adolescents, in both health and disease. Further defining these age-dependent differences will improve our ability to tailor therapies, predict disease course, and distinguish the underlying pathological mechanisms of arthritis across the age spectrum.

## Data Availability

No datasets were generated or analysed during the current study.
